# A review on electronic and optical properties of silicon nanowire and its different growth techniques

**DOI:** 10.1186/2193-1801-2-151

**Published:** 2013-04-10

**Authors:** Mehedhi Hasan, Md Fazlul Huq, Zahid Hasan Mahmood

**Affiliations:** 1 Department of Electrical and Electronic Engineering, Shahjalal University of Science and Technology, Kumargaon, Sylhet, 3114 Bangladesh; 2 Department of Information and Communication Technology, Mawlana Bhashani Science and Technology University, Santash, Tangail, 1902 Bangladesh; 3 Department of applied Physics Electronics and Communication Engineering, University of Dhaka, Dhaka, 1000 Bangladesh

**Keywords:** Silicon Nanowires (SiNWs), Bandgap, Optical absorption, Reflectance, Chemical Vapour Deposition (CVD), Molecular Beam Epitaxy (MBE)

## Abstract

Electronic and optical properties of Silicon Nanowire (SiNW) obtained from theoretical studies and experimental approaches have been reviewed. The diameter dependency of bandgap and effective mass of SiNW for various terminations have been presented. Optical absorption of SiNW and nanocone has been compared for different angle of incidences. SiNW shows greater absorption with large range of wavelength and higher range of angle of incidence. Reflectance of SiNW is less than 5% over majority of the spectrum from the UV to near IR region. Thereafter, a brief description of the different growth techniques of SiNW is given. The advantages and disadvantages of the different catalyst materials for SiNW growth are discussed at length. Furthermore, three thermodynamic aspects of SiNW growth via the vapor–liquid–solid mechanism are presented and discussed.

## Introduction

**Figure 1:**
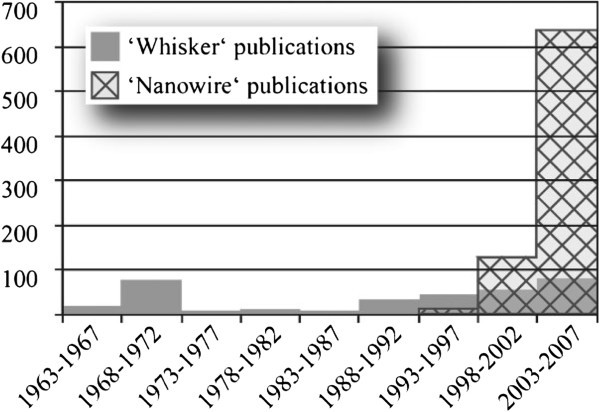
**Histogram of silicon “whisker” and “nanowire” publications. Source: ISI Web of Knowledge (SM).**

Growth of Si whiskers was first reported by Wagner and Ellis as early as 1964 (Wagner & Ellis 1964). Later Givargizov elucidated the growth mechanism of Si whiskers in 1975 (Givargizov 1975). The first report on carbon nanotubes by Iijima in 1991 focused a worldwide exponential increase of research into the carbon based and silicon-based nanomaterials especially carbon nanotubes and SiNWs (Iijima 1991). Subsequently, there were extensive investigations carried out on the synthesis, physical properties, device fabrication and applications of SiNWs. Figure 1 shows a histogram of the number of silicon “whisker” and “nanowire” publications (Schmidt & Wittemann 2009). Due to their novel properties Silicon nanowires (SiNWs) have grabbed such great attentions. It is their unique electrical, optical and mechanical properties that make them to receive such interest. Even more importantly, SiNWs-based nanodevices are compatible with the current Si-based microelectronics industry, and already a number of nanodevices based on SiNWs as building blocks have been demonstrated (Cui & Lieber 2001).

The nanoscale diameter puts the radial dimension of nanowires at or below the characteristic length scale of various interesting and fundamental solid state phenomena: the exciton Bohr radius, wavelength of light, phonon mean free path, critical size of magnetic domains, exciton diffusion length, etc. (Alivisatos 1996; Law et al. 2004). As a result, many physical properties of semiconductors are significantly altered within the confinement of the nanowire surfaces. In addition, their large surface-to-volume ratio allows for distinct structural and chemical behavior as well as greater chemical reactivity. This two-dimensional confinement endows nanowires with unique properties which stray from those of their corresponding bulk material. Second, the large aspect ratio of nanowires intimates their technological application. The one unconstrained dimension can direct the conduction of quantum particles such as electrons, phonons, and photons. This control over various forms of energy transport recommends nanowires as ideal materials from which to manufacture advanced solid-state devices. Moreover, the length of nanowires is normally sufficient to interface with top-down fabrication processes, such as photolithography. As a result, nanowires provide a convenient platform through which researchers may study confined transport phenomena.

## Review

### Properties of Silicon Nanowires

#### Electronic Properties

The small sizes of SiNWs make their electronic and electrical properties strongly dependent on growth direction, size, morphology and surface reconstruction. A well known example is the size dependence of the electronic bandgap width of SiNWs irrespective of wire direction. As the wire diameter decreases, the band gap of the nanowire widens and deviates from that of bulk silicon gradually. Moreover, the orientations of the wire axis and the surface have a great effect on the electronic properties of SiNWs (Aijiang 2007).

**Figure 2:**
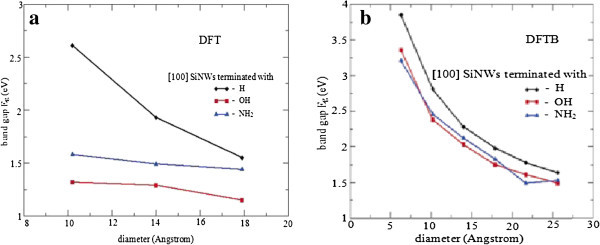
**Band gap as a function of the [100] silicon nanowire diameter for various surface terminations.** (**a**) DFT calculations within GGA-PBE. (**b**) Results from a density-functional tightbinding (DFTB) parameterization.

Michael Nolan et al. theoretically investigated the band gap modification for small-diameter (~1 nm) silicon nanowires resulting from the use of different species for surface termination by density functional theory calculations. Because of quantum confinement, small-diameter wires exhibit a direct band gap that increases as the wire diameter narrows, irrespective of surface termination. Figure 2 shows band gap as a function of nanowire diameter for various surface terminations (Nolan et al. 2007).

Sacconi et al. also investigated the electronic properties of silicon nanowires with several different approaches like the Empirical Tight-Binding (ETB) model, the Linear Combination of Bulk Bands (LCBB) model and Non-Equilibrium Green Function (NEGF) model. They considered both hydrogenated and SiO_2_ terminated silicon surfaces in these models. When the diameter of SiNW is reduced from 3.2 to 1.6 nm, the bandgap of hydrogenated nanowire increases from 1.56 to 2.44 eV. On the other hand, in the case of SiO_2_/SiNW structure, this increase is smaller, since the bandgap goes from 1.50 eV for a 3.2 nm cell, to 1.88 eV for a 1.6 nm cell. This behavior can be expected due to a lower confinement determined by SiO_2_ surrounding SiNW, with respect to the case of simple hydrogen termination.

Effective masses for conduction and valence bands have also been calculated. The effect of an increasing Si thickness on a hydrogen terminated wire is that of reducing the conduction mass, from 0.47 m_o_ to 0.31 m_o_, which is 55% greater than the value of transverse mass in bulk silicon. The effect on the SiO_2_-confined wire is similar; by increasing Si thickness, effective mass decreases from 0.36 m_o_ to 0.29 m_o_ (Sacconi et al. 2007).

The effect of wire thickness of SiNW on conduction valley splitting, hole band splitting, effective masses and transmission has been reported using sp^3^ d^5^ s^*^ model by Yun Zheng et al. They concluded that in the conduction band, valley splitting reduces the averaged mobility mass along the axis of the wire, but quantum confinement increases the transverse mass of the conduction band edge. For the wire thickness range that they have considered, the effective mass at the conduction band edge is at least 35% heavier than that of transverse mass of bulk Si. Quantum confinement has the largest effect on the effective masses in the valence band. The effective mass at the valence band edge is at least six times heavier than that of the bulk. The effective mass of the next highest band is even heavier. Small energy splitting also occurs at the conduction band minimum. For wires greater than 1.54 nm thick, the four bulk valleys which compose the conduction band minimum are split into three energies. The center energy is twofold degenerate roughly evenly split between the lowest and highest energy. The single-band model performs reasonably well at calculating the effective band edges for the 1.54 nm wire (Zheng et al. 2005).

Yi Cui et al. shows that Boron and phosphorus can be used to change the conductivity of SiNWs over many orders of magnitude and that the conductivity of the doped SiNWs respond oppositely to positive (negative) *V*g for boron and phosphorus dopants. Indeed, the *V*g dependence provides strong proof for p-type (holes) doping with boron and n-type (electrons) doping with phosphorus in the SiNWs (Cui et al. 2000).

#### Optical Properties

Bulk Si has an indirect band gap, with the valence band maximum at the Γ point and the conduction minimum at about 85% along the Γ to X direction, and a phonon is required to conserve the momentum in any electronic transition. Remarkably, however, SiNWs grown along most of the crystallographic orientations have a direct band gap, meaning that the maximum of the valence band and the minimum of the conduction band occur at the same point in k-space. This property has allowed to envisage the use of SiNWs as optically active materials for photonics applications (Canham 1990; Guichard et al. 2006).

The possibility of controlling the band gap width is tremendously attractive for optoelectronics applications: not only SiNWs can have a direct band gap, which per se increases the optical efficiency, but its width can in principle be tuned. It is not difficult to imagine, however, that controlling the wire diameter with tolerances within 1–3 nm is a more than challenging task. A simpler route to band gap tuning is controlling the chemical composition and the coverage density of the wire surface. Halogens such as Cl, Br, and I can be used as surface passivation agents instead of H and, while not altering the semiconducting character of the wires, they result in a significant shrinking of band gap (Leu et al. 2006). The strongest reduction of the band gap is provided by I, followed by Br and Cl, in the opposite order of the bonding strength of these species and SiNWs. Interestingly, the surface coverage is a further degree of freedom and one can span all the band gap values between a H- and halogen-passivated wire by varying the H:halogen ratio. Also, increasing the halogen surface concentration the band edge states, concentrated in the wire core in presence of H-passivation, progressively spread to the surface.

Analogous results have been reported for OH and NH2 (Aradi et al. 2007; Nolan et al. 2007). It should be noted that the passivating species do not contribute significantly to the states close to the band edges, so that the reduction of the gap is not caused by the introduction of additional bands. It rather comes from the hybridization of the valence band states with the frontier orbitals of the different passivating functional groups that cause a significant band gap reduction relative to H-passivated wires.

These results indicate that the band gap width in SiNWs can be tailored not only by controlling the wire diameter, but also by an appropriate choice of the surface termination.

**Figure 3:**
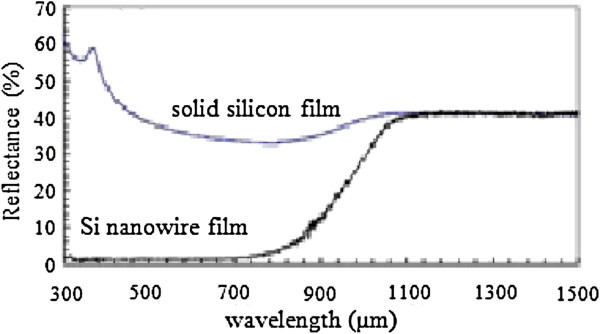
**Total reflectance data from integrated sphere measurements for an 11 μm thick solid Si thin film and nanowire film on glass substrate (Tsakalakos et al.**^2007^**)**

The broadband optical absorption properties of silicon nanowire (SiNW) films have been measured and found to be higher than that of solid thin films of Si of equivalent thickness. The observed behavior is adequately explained by light scattering and light trapping though some of the observed absorption is due to a high density of surface states in the nanowires films, as evidenced by the partial reduction in high residual sub-bandgap absorption after hydrogen passivation. The reflectance of the solid film shows typical behavior what expected for silicon, whereas the reflectance of the nanowire film is less than 5% over the majority of the spectrum from the UV to the near IR and begins to increase at ~700 nm to a values of ~41% at the Si band edge (1100 nm), similar to the solid film sample. It is clear that the nanowires impart a significant reduction of the reflectance compared to the solid film. Figure 3 shows comparative reflectance of solid silicon and Si nanowires (Tsakalakos et al. 2007).

**Figure 4:**
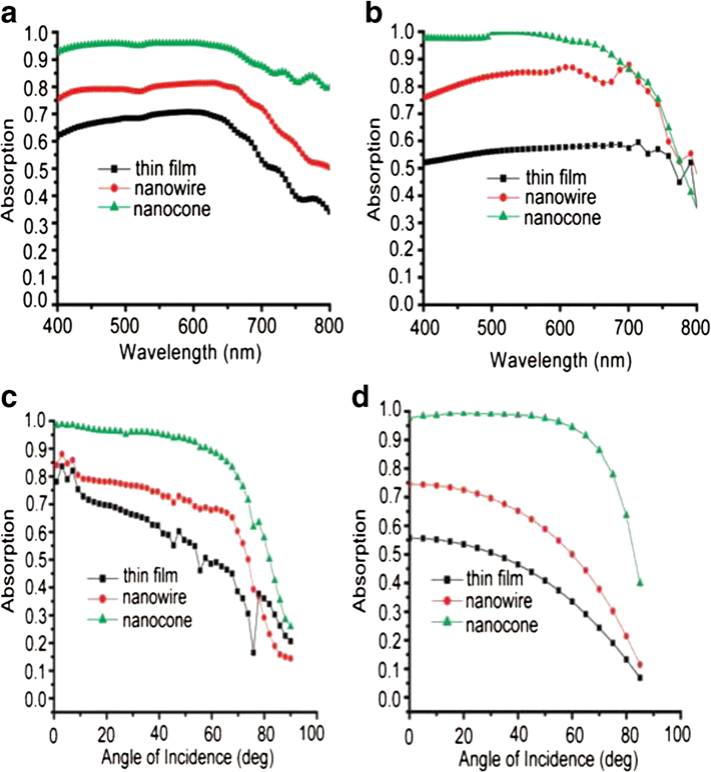
**Value of absorption on samples with a-Si:H thin film, NW arrays and NC arrays (a) Measured, (b) Calculated over a large range of wavelengths at normal incidence; (c) Measured (d) Simulated for different angle of incidence (at wavelength*λ*= 488 nm) (Zhu et al.**^2009^**).**

Fabrication of Si:H nanowires (NWs) and nanocones (NCs), using an easily scalable and IC-compatible process has been reported by Jia Zhu *et al*. They have shown that Si:H nanostructures display greatly enhanced absorption over a large range of wavelengths and angles of incidence due to suppressed reflection. More than 90% of light is absorbed at angles of incidence up to 60° for a-Si:H NC arrays, which is significantly better than NW arrays (70%) and thin films (45%). In addition, the absorption of NC arrays is 88% at the band gap edge of a-Si:H, which is much higher than NW arrays (70%) and thin films (53%). Figure 4 summarized the results of different structures for different condition (Zhu et al. 2009).

#### Methods of Fabrication of Nanowires

Many techniques, including both top-down and bottom-up approaches, have been developed and applied for the synthesis of Nanowires. Vapor–Liquid–Solid (VLS) Mechanism, Chemical Vapor Deposition (CVD), Evaporation of SiO, Molecular Beam Epitaxy (MBE), Laser Ablation and Electroless metal deposition and dissolution (EMD) have been discussed here.

#### Vapor–Liquid–Solid (VLS) Mechanism

**Figure 5:**
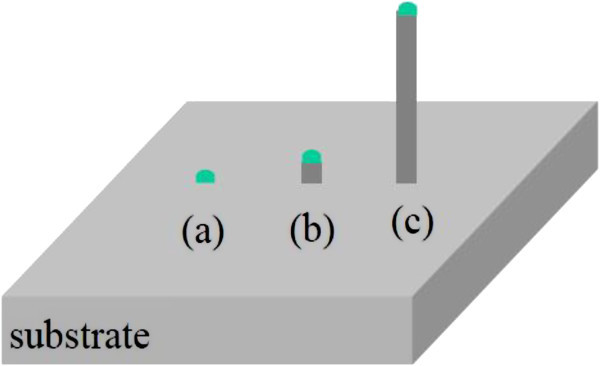
**Schematic of the VLS growth mechanism (a) Catalytic liquid alloy (b,c) Successive growth of nanowire.**

The VLS mechanism, first proposed by Wagner and Ellis (Wagner & Ellis 1964) in the mid-1960s, is the key mechanism for silicon-wire growth. Their proposed VLS mechanism is based on two observations: that the addition of certain metal impurities is an essential prerequisite for growth of silicon nanowires in experiments and that small globules of the impurity are located at the tip of the wire during growth. From this, Wagner and Ellis deduced that the globule at the wire tip must be involved in the growth of the silicon wires by acting “as a preferred sink for the arriving Si atoms or, perhaps more likely, as a catalyst for the chemical process involved” (Wagner & Ellis 1964). When Au, for example, is deposited on silicon substrate and this substrate is then heated to temperatures above about 363°C, small liquid Au–Si alloy droplets will form on the substrate surface. Exposing such a substrate to a gaseous silicon precursor, such as silicon tetrachloride (SiCl_4_) or silane (SiH_4_) precursor molecules will crack on the surface of the Au–Si alloy droplets, whereupon Si is incorporated into the droplet. The silicon supply from the gas phase causes the droplet to become supersaturated with Si until silicon freezes out at the silicon/droplet interface. The continuation of this process then leads to the growth of a wire with the alloy droplet riding atop the growing wire (Wagner & Ellis 1964) (Figure 5).

#### Chemical Vapor Deposition (CVD)

**Figure 6:**
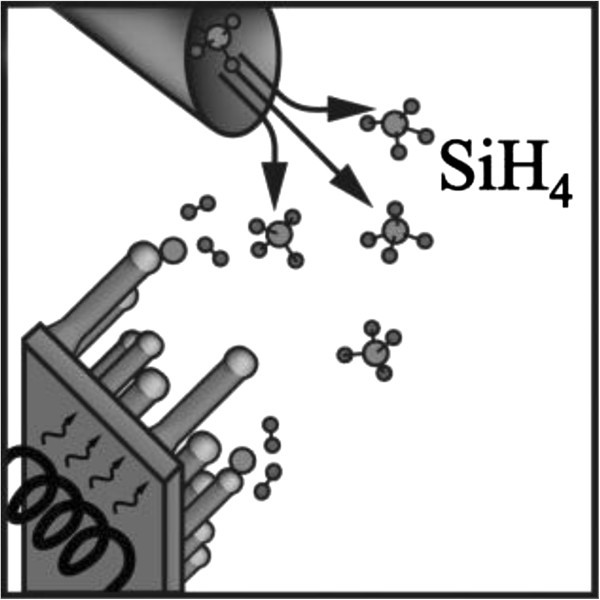
**Schematics of experimental setup for nanowire growth using CVD method.**

In CVD, a volatile gaseous silicon precursor, such as silane (SiH_4_) or silicon tetrachloride (SiCl_4_), serves as the silicon source. It is transported to the deposition surface at which the precursor reacts, and is cracked into its constituents as depicted in Figure 6.

Originally, CVD was devised for the deposition of high-purity films. Contaminations such as gold particles, however, were found to cause anisotropic growth of silicon, that is, the growth of silicon wires. CVD allows epitaxial growth of SiNWs, with the growth velocity varying from about 10^-2^ to 10^3^ nm min^-1^, (Kodambaka et al. 2006; Nebol’sin et al. 2005) depending on temperature and type of Si precursor used. Furthermore, CVD offers broad possibility of modifying the properties of the silicon wires in a controlled fashion. A variety of derivatives of CVD methods exist. These can be classified by parameters such as the base and operation pressure or the treatment of the precursor. Since silicon is known to oxidize easily if exposed to oxygen at elevated temperatures, it is crucial to reduce the oxygen background pressure in order to be able to epitaxially grow uniform silicon nanowires. In particular, when oxygen-sensitive catalyst materials are used, it turns out to be useful to combine catalyst deposition and nanowire growth in one system, so that growth experiments can be performed without breaking the vacuum in between (Wang et al. 2006). In any case, it is useful to lower the base pressure of the CVD reactor down to high or even ultrahigh vacuum, which reduces unwanted contamination and enables growth at lowered temperatures (Akhtar et al. 2008). The pressures during growth mainly depends upon the gaseous silicon precursor and its cracking probability at the catalyst surface. Growth with disilane, Si_2_H_6_, for example, can—but must not—be carried out at extremely low partial pressures of around 10^-6^ mbar (1 bar=10^5^Pa). These low growth pressures allow the combination of CVD with transmission electron microscopy (TEM), enabling in situ observation of the nanowire growth (Hofmann et al. 2008). In contrast to that, silane partial pressures required for wire growth are about five orders of magnitude higher. By modifying the precursor before reacting with the sample surface, the temperature budget of the substrate can be lowered. In cases where the thermal load is critical or where a high supersaturation of the droplet is necessary, nanowire growth can be enhanced using plasma-enhanced CVD (PECVD) (Hofmann et al. 2003; Sharma et al. 2004; Iacopi et al. 2007). Another advantage of CVD as a bottom-up synthesis method is its variability concerning the intended wire size. Wire diameters range from below 10 nm (Cui et al. 2001) up to several hundred micrometers (Wagner & Ellis 1964). Since surface diffusion only plays a minor role in CVD, the length of the wires can also be tuned accordingly by simply extending or decreasing the growth time. Thus, to summarize, a large range of length and diameter configurations can be fabricated (Park et al. 2008). With CVD, not only the wire size but also its properties can be modified.

#### Evaporation of SiO

**Figure 7:**
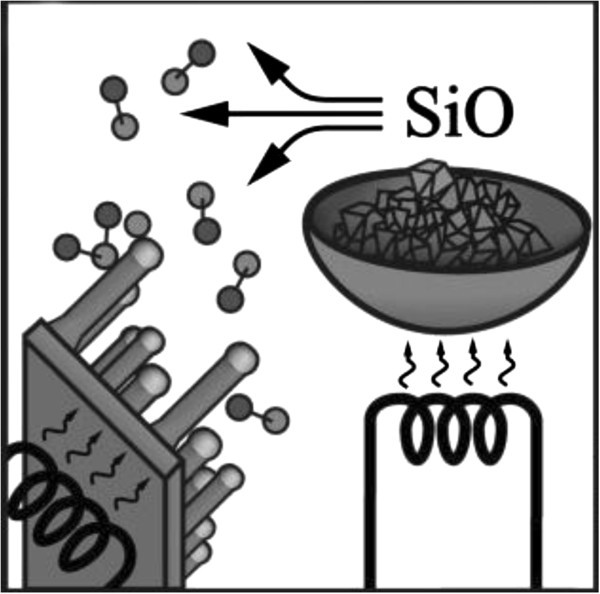
**Schematics of experimental setup for silicon nanowire growth by evaporation of SiO.**

A cost-effective method to produce silicon nanowires on a large scale is to evaporate solid silicon monoxide, SiO (shown in Figure 7).

A two-zone tube furnace connected to an inert gas supply and small amount of SiO granulate are the basic ingredients for the synthesis of silicon nanowires. Crucial for growth is a temperature gradient from about 1350 to 900°C along the tube of the furnace. SiO is evaporated at the hotter end of the tube, flows with the gas stream to the cooler part, where it undergoes a disproportionation reaction into Si and SiO_2_, thereby forming the nanowires (Pan et al. 2001). In principle, two different growth methods are possible: growth with and without metal catalyst. Growth assisted by the presence of a metal catalyst is relatively rapid (Gu et al. 2000). Consistent with the concept of VLS growth, the diameters are determined by the size of the catalyst particle, although the interplay between the nanowire and the catalyst droplet seems to be more complex compared to normal CVD growth. As a consequence of the disproportionation reaction, the diameter ratio between crystalline core and amorphous shell remains approximately constant (Kolb et al. 2004). The second growth mode, metal-catalyst-free growth, has been originally proposed for growth via laser ablation (Wang et al. 1998), where it was observed that nanowires can be catalyzed by silicon dioxide (Wang et al. 1999). Remarkable about this oxide-assisted growth (OAG) is that SiO_2_-containing targets clearly raise the yield of the final amount of silicon nanowires compared to pure silicon targets or mixed silicon–metal targets (Wang et al. 1998). By carrying out the growth process over several hours, one can obtain millimeter-long crystalline silicon nanowires with varying diameters from about 5 nm to 100 nm, covered by an amorphous shell of up to several 10 nm (Shi et al. 2000; Zhang et al. 2000; Shi et al. 2005).

#### Molecular Beam Epitaxy (MBE)

**Figure 8:**
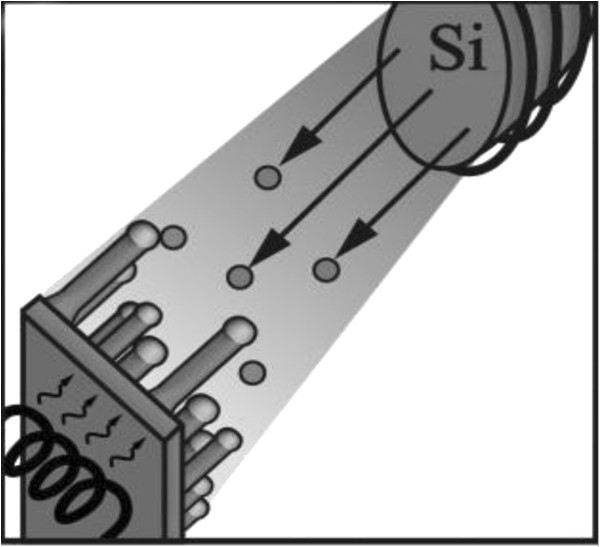
**Schematics of experimental setup for silicon nanowire growth by MBE.**

In MBE, a solid high-purity silicon source is heated until Si starts to evaporate. Figure 8 schematically depicts an MBE setup.

A directional gaseous beam of silicon atoms is aimed at the substrate, on which the atoms adsorb and crystallize. To reduce contamination, the base pressure of an MBE system is usually kept at ultrahigh vacuum, allowing to monitor the growth using Reflection High-Energy Electron Diffraction (RHEED) (Werner et al. 2006) or other surface sensitive examination methods. Similar to CVD, MBE was initially designed for epitaxial layer-by-layer deposition only. Yet, metal contamination was also found to cause silicon-wire growth in this case. Differing from CVD, no precursor gas is cracked at the surface of the liquid metal–silicon alloy. Therefore, the latter cannot be treated as a classical catalyst anymore. In MBE, two silicon fluxes govern wire growth. First, the direct flux of silicon from the silicon source; and second, the flux of diffusing silicon adatoms from the silicon substrate surface. The nanowires produced by MBE—usually grown on Si(111) substrates—are epitaxial and <111> oriented. MBE offers excellent controllability in terms of the incoming flux, such that doped wires (Kanungo et al. 2008) or heterostructures (Zakharov et al. 2006) can be grown by switching between evaporation sources. One disadvantage of MBE, however, is that the method is limited with respect to the minimally possible Si-nanowire diameter. Only nanowires with diameters greater than about 40 nm can be obtained (Shi et al. 2005; Schubert et al. 2004) which seems to be a consequence of the Gibbs–Thomson effect and the fact that only small Si supersaturations are achievable by MBE. Another disadvantage of MBE is the low nanowire growth velocity of just a few nanometers per minute (Schubert et al. 2004).

#### Laser Ablation

**Figure 9:**
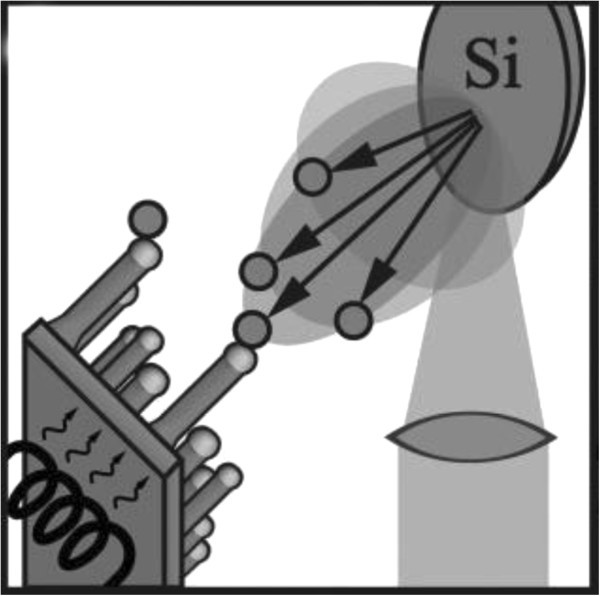
**Schematics of experimental setup for silicon nanowire growth by Laser Ablation method.**

The silicon nanowires produced by laser ablation differ in many aspects from the MBE grown whiskers. One can easily obtain large quantities of ultrathin nanowires with high aspect ratios (Zhang et al. 1998; Zhou et al. 1998). As schematically displayed in Figure 9, a high-power pulsed laser ablates material from a mixed Si–catalyst target, which is placed in a tube furnace held at high temperatures and purged with an inert gas.

The silicon material ablated from the target cools by colliding with inert-gas molecules, and the atoms condense to liquid nanodroplets with the same composition as the target (Morales & Lieber 1998). Thus, these nanoparticles contain both Si and the catalyst material. According to the VLS mechanism, silicon nanowires start to grow once the catalyst gets supersaturated with silicon and proceeds as long as the catalyst nanoparticles remain liquid. The advantages of laser-ablated nanowire production are manifold. First, there is no need for a substrate. Second, the composition of the resulting nanowires can be varied by changing the composition of the laser target. By adding, for example, SiO_2_ to the target, single-crystalline silicon nanowires with varied amorphous SiO_x_ shell thicknesses can be obtained in a single processing step (yang et al. 2004) with silicon-core diameters as low as 5 nm and varying shell thicknesses of about 10 nm. Due to the high growth temperatures, catalyst metals such as Fe, possessing a high eutectic temperature, can be used. The resulting nanowire growth velocities are typically of the order of micrometers per minute (Zhang et al. 1998; Morales & Lieber 1998). The radii of the nanowires not only depend on the type of metal catalysts used but also on the gases that are streamed through the furnace, such as H_2_ , He, or N_2_ (Zhang et al. 1999).

#### Electroless metal deposition and dissolution

**Figure 10:**
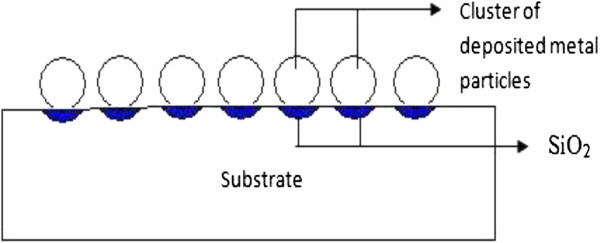
**Oxidation of silicon surface under deposited metal.**

Electroless deposition, a non-galvanic type of deposition method that involves several simultaneous reactions in an aqueous solution, which occur without the use of external electrical power. The most common electroless deposition is nickel, silver or gold particle deposition. First step of silicon nanowire synthesis using this process is to deposit metal particle like Au, Ag or Cu on silicon substrate. These noble metals would attract electrons from the silicon and facilitate Si oxidation. Figure 10 shows the oxidation of silicon surface under deposited metal.

**Figure 11:**
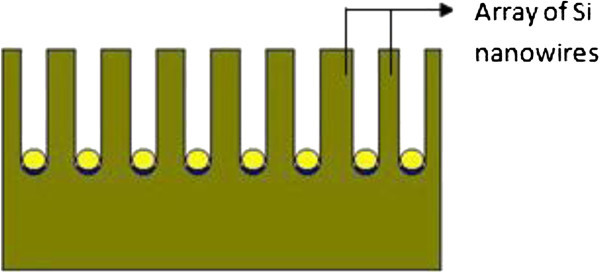
**Formation of silicon nanowires by electroless metal deposition.**

Generally, the etching of a cleaned Si wafer proceeds very slowly in aqueous HF/Fe(NO_3_)_3_ solution at low temperature. However, Si etching occurs rapidly when Si substrates covered with Ag/Au-nanoparticle films are immersed in HF/Fe(NO_3_)_3_ solution at room temperature. Formation of SiNW arrays due to the further sinking of the Ag particles, and longitudinal and lateral dissolution of bulk Si (shown in Figure 11) (Peng et al. 2006).

## Conclusions

In summary, we have seen that there are significant changes in electronic and optical properties of silicon nanowires than those of bulk. The change of some properties depends on the size and shape of the nanostructures. It suggests that, careful production of nanowires of desired size and shape would make it possible to manipulate properties like bandgap, effective mass and optical absorption. It also has been seen that silicon nanowires can be produced by different growth methods. Au and Ag are the most popular catalyst material for silicon nanowire synthesis.
